# The sound of stress recovery: an exploratory study of self-selected music listening after stress

**DOI:** 10.1186/s40359-023-01066-w

**Published:** 2023-02-10

**Authors:** Krisna Adiasto, Madelon L. M. van Hooff, Debby G. J. Beckers, Sabine A. E. Geurts

**Affiliations:** 1 Behavioural Science Institute, Radboud University, Nijmegen, The Netherlands; 2 Faculty of Psychology, Open Universiteit, Heerlen, The Netherlands

**Keywords:** Music, Stress, Recovery, Mood, Emotions, Audio features, Dynamics, Tempo, Spotify

## Abstract

**Background:**

Empirical support for the notion that music listening is beneficial for stress recovery is inconclusive, potentially due to the methodological diversity with which the effects of music on stress recovery have been investigated. Little is presently known about which recovery activities are chosen by individuals for the purpose of stress recovery, and whether audio feature commonalities exist between different songs that are selected by individuals for the purpose of stress recovery. The current pre-registered study investigated whether audio feature commonalities can be extracted from self-selected songs for the purpose of stress recovery. Furthermore, the present study exploratorily examined the relationship between audio features and participants’ desired recovery-related emotions while listening and after listening to self-selected music.

**Methods:**

Participants (*N* = 470) completed an online survey in which they described what music they would listen to unwind from a hypothetical stressful event. Data analysis was conducted using a split-sample procedure. A *k*-medoid cluster analysis was conducted to identify audio feature commonalities between self-selected songs. Multiple regression analyses were conducted to examine the relationship between audio features and desired recovery emotions.

**Results:**

Participants valued music listening as a recovery activity to a similar extent as watching TV, sleeping, or talking to a significant other. Cluster analyses revealed that self-selected songs for the purpose of stress recovery can be grouped into two distinct categories. The two categories of songs shared similarities in key, loudness, speechiness, acousticness, instrumentalness, liveness, musical valence, tempo, duration, and time signature, and were distinguished by danceability, energy, and mode. No audio features were significantly associated with participants’ desired recovery emotions.

**Conclusions:**

Although a comprehensive portrait of the relationship between audio features and stress recovery still warrants further research, the present study provides a starting point for future enquiries into the nuanced effects of musical audio features on stress recovery.

**Supplementary Information:**

The online version contains supplementary material available at 10.1186/s40359-023-01066-w.

## Background

Adequate day-to-day stress recovery is essential to maintain our psychological and physiological well-being in the long-term [1, 2]. This can be understood from the perspective of the Effort-Recovery (ER) model [3], which posits that exerting effort to deal with demands or stressors will induce physiological (e.g., increased heart rate, blood pressure, and cortisol) and psychological (e.g., increased anxiety, distress, fatigue, and depressed mood) load reactions in individuals [2, 4]. The ER model further states that in the absence of demands or stressors, a recovery process occurs in which these load reactions are alleviated, allowing individuals to adequately deal with subsequent demands or stressors. However, when demands or stressors are continually present, an accumulative process may be started that is detrimental in the long-term: as sufficient recovery cannot occur, individuals must exert compensatory effort to deal with new demands and stressors, which in turn requires further recovery [1]. Studies have linked chronic, insufficient stress recovery to the incidence of various stress-related disorders, including cardiovascular disease, burnout, and depression [1, 2, 5]. As such, it is important to investigate how effective recovery from stress can be promoted on a day-to-day basis.

Music listening stands to be a promising strategy to facilitate adequate stress recovery. Indeed, the previous decade has seen growing interest in the potential of music-based interventions as an efficient and accessible means of promoting sufficient stress recovery [6, 7]. However, despite the popular notion that listening to music may help us feel better after stressful situations [8, 9], there is limited information on whether individuals do make use of music listening as an activity to unwind from stress, especially compared to other recovery activities such as sleep, physical activity, or meditation [2, 10]. Further, empirical support for the beneficial effect of music listening on stress recovery, particularly in healthy individuals, is inconclusive [7]. Studies suggest that listening to music is more effective than silence or an audio control in promoting heart rate [11], blood pressure [12], anxiety [13, 14], and mood [14–16] recovery following a stressor, but a comparable number of studies do not report similar findings [17–20]. This discrepancy may be a consequence of the high heterogeneity that characterizes present research on music listening and stress recovery. Most notably, the breadth of strategies with which studies have selected musical stimuli for use in experimental studies, such as sampling relaxation tapes [12], consulting experts [19], and allowing participants to choose their own songs (i.e., self-selection) [21], renders it difficult to contrast findings between studies [7, 8]. The present study addresses these unresolved matters to determine which music is most effective for the purpose of stress recovery.

From a theoretical perspective, listening to music is thought to facilitate stress recovery through positive emotional experiences. This assumption is rooted in evidence that positive emotions activate the brain’s ‘pleasure and reward center,’ releasing hormones such as serotonin and dopamine, which help downregulate the physiological and psychological load reactions that are part of the stress response [22]. Similarly, the broaden-and-build theory of positive emotions [23] states that positive emotional experiences may counteract the negative emotions elicited by a stressor, and in doing so help prevent the detrimental consequences of stress [24]. Further, the broaden-and-build theory postulates that positive emotions encourage novel, exploratory thoughts and behaviours, which over time enhances an individual’s ability to deal with demands or stressors [25]. Indeed, studies have demonstrated that positive emotions interact with our stress systems in functionally meaningful ways, such as by lowering heart rate, blood pressure, and cortisol [26, 27], significantly lowering the incidence of cardiovascular disease in the long-term [28].

Given the supposed role of positive emotions in the stress recovery process, it is reasonable to assume that music has recovery potential under the condition that it elicits a favourable emotional response. Based on this assumption, the field of music emotion recognition (MER) provides hints to which songs may be most beneficial for stress recovery. Music emotion recognition studies have related different (combinations of) musical audio features, such as tempo (i.e., how fast a song is), pitch (i.e., the value of a particular note or sound), and timbre (i.e., the overall quality of a song) to the valence (i.e., whether an emotion is positive, with higher valence representing more positive emotions) and arousal components of self-reported musical emotions [7, 29, 30]. For example, tempo is positively related to emotional valence and emotional arousal, such that songs with faster tempi are associated with emotions with more positive valence and higher arousal [30–32]. Similarly, pitch, timbre, and loudness (i.e., perceived sound pressure) were positively and linearly correlated with emotional arousal, such that songs with lower pitch, softer timbre, and lower sound pressure are associated with emotions that are lower in arousal [30, 32].

Despite this, the term ‘musical emotions’ in MER refers to emotions that are ‘perceived’ while listening to music (i.e., a song’s emotional content), and provides little attention to emotions that are ‘felt’ after listening to music (i.e., an individual’s emotional outcome) [33–35]. This distinction may be particularly important in the association between music listening, emotional experiences, and stress recovery, as it is likely the emotional outcome of music listening that influences the stress recovery process. Indeed, studies have suggested that even sad songs may help individuals feel better after stress [36–38]. Furthermore, studies have demonstrated that individuals who listened to music that was congruent with their emotional self-regulation goals (i.e., attempts at changing or maintaining emotional states) experienced emotions with higher emotional valence and lower emotional arousal, compared to individuals who listened to music that was incongruent with their goals [39–44].

Thus, in line with the notion that music listening is a personal experience [45, 46], the audio features of songs that individuals select for the purpose of stress recovery are likely heterogeneous. Yet, it is currently difficult to comprehensively examine this heterogeneity, as studies that have investigated the effects of self-selected music on stress recovery often do not report the specific songs that are selected by participants [e.g., 15, 21. Despite this heterogeneity, it is reasonable to assume that self-selected stress recovery songs may share similarities in specific (combinations of) audio features, which allow these self-selected songs to elicit favourable emotional experiences. As such, exploring the relationship between musical audio features, perceived emotions, and felt emotions to self-selected stress recovery songs may be a promising approach to determine which music works best to facilitate stress recovery.

In summary, the present study further considers the potential of music listening as a strategy to promote adequate stress recovery. In an exploratory manner, the present study aims to investigate: (a) which recovery activities are chosen by individuals for the purpose of stress recovery; (b) audio feature commonalities between self-selected stress-recovery songs; and (c) the association between different audio features and participants’ emotional responses to music listening.

## Method

### Participants

Participants were recruited over a period of three months (August – October 2020) through SurveyCircle (https://www.surveycircle.com) and social media posts. Participants were eligible to take part in the study if they were 18–65 years old and were able to access the survey link with their computers, laptops, tablets, or smartphones. At the end of our data collection period, responses from 653 participants were obtained. From these responses, 183 were removed due to participants declining or not responding to any of the survey items. Our final data set consisted of responses from 470 participants (*m*_*age*_ = 28.2, *SD*_*age*_ = 7.76, *range* = 18–63), of which 145 (30.85%) reported being proficient with a musical instrument, and 166 (35.32%) reported having had formal musical training. Participants were predominantly nationals of a European (48.06%) or Asian country (44.89%). A complete list of participant nationalities can be found in Additional file 1: Appendix A.

### Measures

To obtain a portrait of participants’ choice of stress recovery activities, self-selected recovery songs, and emotional responses to music listening, our pre-registered, exploratory cross-sectional study employed a survey that included items adapted from existing, validated measures. Prior to our data collection period, the survey was pilot tested for comprehension and reliability. The survey was distributed online to facilitate the collection of large amounts of data within the planned recruitment period. The survey contained the following measures:

#### Background information

##### Age and nationality

Participants reported their age (in years and months) and nationality by typing in their answer in separate text boxes.

##### Musical proficiency

Studies suggest that musical proficiency may influence how individuals experience and process musical emotions [47]. Thus, adapting from Chin and Rickard’s music USE (MUSE) questionnaire [48], participants reported whether they played a musical instrument (“yes” or “no”). If “yes” was selected, participants reported the instrument they played by typing in their answer in a text box and reported how long (in years and months) they have been playing that instrument. When participants played more than one musical instrument, they were asked to provide the above information for the instrument they have played the longest. Additionally, participants reported whether they have received any formal musical training (i.e., studying music at a university, academy, conservatorium, or through private or group lessons; “yes” or “no”). If “yes” was selected, participants reported the duration (in years and months) of their musical training. The MUSE questionnaire has previously been used to reliably assess instrument playing and musical training [49].

#### Choice of recovery activities

Participants rated their likelihood (0: highly unlikely; 100: highly likely) of performing certain activities when they wished to recover from stress. Based on reviews of various stress recovery activities [2, 10], participants were asked to rate the following activities: light physical exercise, sports/heavy physical exercise, reading a book, watching a TV series, watching a movie, talking to friends, talking to a partner/significant other, talking to family, playing a musical instrument, listening to self-selected music, listening to the radio, sleeping, eating, drinking alcoholic beverages, praying, meditating, mindfulness. To standardize their understanding of stress recovery, participants read the following information before rating each recovery activity: “In one way or another, we all experience stress in our everyday lives. To remain healthy in the long-term, it is important for us to both physically and mentally unwind after experiencing stressful events. This process of unwinding is called stress recovery.”

#### Choice of music

Regardless of their choice of recovery activities, participants described the music they would most want to listen to after a stressful situation. This description included:

##### Musical genre

Participants selected which genre the music should be. Based on common genres in popular music [50], participants could select one or more of the following genres: ambient, blues, classical, electronic, jazz, metal, pop, punk, reggae, rock, R&B, ska, movie/series soundtracks. Given the definition of musical genres tends to be somewhat arbitrary [51], participants were also given the option of specifying a genre themselves.

##### Perceived tempo

Given the positive association between tempo and an emotion’s valence and arousal components [30, 31], participants indicated how fast the music should be. Participants provided a descriptive estimate of their music’s tempo (“fast/upbeat”, “moderate”, or “slow/downbeat”) as self-reported numerical estimates of tempo (e.g., “115 bpm”, “101–120 bpm”) often do not match actual tempo rates [52].

##### Dominant instrument

Given the positive association between timbre and an emotion’s arousal component [30–32], participants selected which instrument should be most prominent. Different instruments produce different patterns of frequencies, resulting in different sound qualities (i.e., timbres). Participants could select one or more of the following instruments: acoustic guitar, electric guitar, bass, cello, drums, flute, organ, piano, samples, saxophone, synthesizer, trumpet, violin, and could specify an additional instrument.

##### Lyrical content

Studies have suggested that lyrical content may influence emotional responses to music [53]. Thus, participants indicated whether the music should contain lyrics (“the song should contain lyrics” or “the song should be instrumental”). If “the song should contain lyrics” was selected, participants described what the lyrics should be about, by typing in their answer in a text box.

##### Emotional responses

Participants reported the emotions they wished to experience for the purpose of stress recovery. Following Gabrielsson [33], we distinguished between ‘perceived emotions’ (i.e., emotions experienced while listening to music) and ‘felt emotions’ (i.e., emotions experienced after listening to music) to music [34]. Participants reported their perceived and felt emotions in two steps. First, participants named their desired emotion by typing in their answer in a text box. Then, participants indicated where the emotion was located on a grid (600 × 600 pixels), which was based on the circumplex model of affect [54, 55]. The x-coordinate represented the emotion’s valence (0: negative–600: positive), while the y-coordinate represented the emotion’s arousal (0: low energy–600: high energy). The midpoint of the grid, representing neutral valence and arousal, was x: 300, y: 300. This grid-based measure was chosen as a more embodied method of measuring emotional valence and arousal, and has previously been used to continuously and accurately measure emotional responses to music listening [54, 56].

##### Reasons for listening to music

Since individuals may adopt music listening as a form of emotional self-regulation [39, 57], participants reported the reasons why music listening would help them unwind from stress. Participants could select one or more of the following reasons [40]: it helps me feel relaxed, it makes me feel more positive, it helps me put things in perspective, it helps me think of solutions, it helps me deal with my emotions, it distracts me from negative thoughts, it reminds me of good memories, it makes me feel understood. Participants were also given the option of specifying their own reason.

##### Song title

Participants reported the title(s) of specific songs that fit the music description they have provided, by typing in the artist’s name and song title in a text box.

#### Audio features

Musical audio features are leveraged extensively in music recommender systems, such as those utilized by music streaming platforms such as Spotify. This provides researchers convenient access to audio feature data, without the need to run complex music information retrieval procedures themselves [58, 59]. Audio feature information for each reported song title was obtained via the Spotify application programming interface (API; https://developer.spotify.com/). The Spotify API allows researchers to access various metadata about artists, albums, and songs from the Spotify Data Catalogue (SDC), which include proprietary audio features (Spotify audio features) that have been extracted from combinations of more conventional features such as tempo, pitch, and timbre [46]. Table 1 provides an overview of Spotify audio features that were extracted for each song.

**Table 1:** Extracted spotify audio features for every song

Audio feature	Description
Key	The musical key of a song. In the SDC, keys are mapped using standard pitch class notation, such that ‘0’ corresponds to the C key, ‘1’ to C^#^/D^b^, ‘2’ to D, etc
Mode	The modality of a song, which represents the type of scale from which the melodic content of a song is derived. Typically, songs in minor mode (0) are considered ‘sad’, while songs in major mode (1) are considered ‘happy’
Time signature	The number of beats (3–7) within each measure of a song, corresponding to time signatures ‘3/4’, ‘4/4’, ‘5/4’, etc
Acousticness	Indicates whether a song was played acoustically: where classical instruments are used instead of electric or electronic instruments. Acousticness ranges from 0.0 to 1.0, where higher values indicate that more of a song is acoustic
Danceability	Indicates whether a song is suitable to dance to. Danceability ranges from 0.0 to 1.0, where higher values indicate that songs are more danceable
Energy	A measure of a song’s intensity and activity. Energy ranges from 0.0 to 1.0, where higher values indicate that songs are more energetic (e.g., faster, louder, noisier)
Instrumentalness	An estimate of whether a song contains vocals. Instrumentalness ranges from 0.0 to 1.0, where values above 0.5 indicate that songs contain little to no vocals (i.e., instrumental)
Liveness	An estimate of whether a song is a live performance in the presence of an audience. Liveness ranges from 0.0 to 1.0, where values above 0.8 indicate that songs are likely to have been a live performance
Loudness	The overall loudness of a song in decibels (dB)
Speechiness	An estimate of the presence of spoken words in a song. Speechiness ranges from 0.0 to 1.0, where values below 0.33 indicate that tracks contain music only, values between 0.33 and 0.66 indicate tracks contain both music and spoken words, and values above 0.66 indicate that tracks consist entirely of spoken words (e.g., podcasts, interviews)
Musical valence	An estimate of the overall positiveness of a song. Valence ranges from 0.0 to 1.0, where higher values indicate that songs feel more positive (e.g., happy, cheerful, euphoric)
Tempo	An estimate of the overall tempo of the song in beats per minute (BPM)
Duration	The duration of the song in seconds

### Procedure

Upon accessing the study link, participants were redirected to a web page with information about the study. Participants were also informed that their responses would be anonymized, and that they could withdraw their participation at any point during the study. Participants were then asked to provide informed consent prior to beginning the study. After providing consent, participants took part in the online survey, in which they reported their background information (age, nationality, musical proficiency), choice of recovery activity, and choice of music (musical genre, perceived tempo, dominant instrument, lyrical content, emotional responses, song titles, reasons for listening to music). After completing the online survey, participants were thanked for their participation, and given the option of obtaining an infographic summarizing group-level results of the study. Based on survey metadata, completing the study required approximately ten minutes.

### Data analysis

Data analysis was conducted in R [60]. To investigate participants’ recovery activity choices, a within-subjects ANOVA was conducted to compare mean scores of each recovery activity. Next, frequency scores for items measuring participants’ reasons for listening to music after stress, characteristics of self-selected songs, and desired emotional responses to music were summarized and described.

#### Split-sample procedure

Next, a split-sample procedure was implemented to investigate our remaining exploratory research aims. Split-sample procedures minimize false discovery and family-wise error rates by splitting sample data into exploratory and confirmatory subsets [61, 62]. Analyses are first conducted in the exploratory subset, following which identified models are retested in the confirmatory subset to check whether findings in the exploratory subset are reproducible in the confirmatory subset [62]. Given the exploratory nature our study aims, we felt this procedure would help ensure that our findings were reliable and generalizable. Following procedure in Anderson and Magruder [62], we created an exploratory subset by randomly sampling half of our data using the ‘sample’ function from package ‘dplyr’ [63], and reserved the remaining half of our data as a confirmatory subset. Next, we conducted the following analyses:

##### Self-selected stress recovery songs and their audio feature commonalities

To determine audio feature commonalities between self-selected recovery songs, Spotify audio features for each song title were extracted using functions from the package ‘spotifyr’ [64]. Next, in the exploratory subset, we conducted a *k*-medoid cluster analysis [65, 66] using the ‘pam’ function of package ‘cluster’ [67]. Spotify audio features key, mode, time signature, acousticness, danceability, energy, instrumentalness, liveness, loudness, speechiness, musical valence, and tempo were used as clustering variables. We chose to partition around medoids, rather than means, as our clustering variables were a mix of both categorical and continuous variables [65]. The optimal number of clusters ‘*k*’ was estimated by calculating silhouette widths for *k* = 2–10, selecting the highest width as the optimal value for ‘*k*'. Finally, we attempted to reproduce the results of the exploratory subset by clustering data in the confirmatory subset using the same clustering variables, clustering algorithm, and number of clusters.

##### Song audio features and desired emotional responses

To examine the relation between Spotify audio features and participants’ desired emotional responses to self-selected music listening, we conducted multiple regression analyses in our exploratory data subset using Spotify audio features (key, mode, time signature, acousticness, danceability, energy, instrumentalness, liveness, loudness, speechiness, musical valence, tempo) as predictors and the following outcomes: (a) perceived emotion valence (PEV); (b) perceived emotion arousal (PEA); (c) felt emotion valence (FEV); (d) felt emotion arousal (FEA). PEV and PEA were added as predictors to the regression equations with FEV and FEA as outcome, respectively. We then attempted to reproduce the results of the regression analyses in the exploratory subset by estimating the same regression equations in the confirmatory subset.

## Results

### Descriptive statistics

#### Participants’ choice of recovery activity

Table 2 provides a summary of means and standard deviations of self-reported scores for each recovery activity. A within-subjects ANOVA suggested there was a significant mean difference among recovery activities (*F*[11,5341] = 162.70, *p* < 0.001). To determine the extent to which music listening was chosen as a recovery activity, mean differences were further explored with Bonferroni-corrected pairwise comparisons (Table 2). Mean scores for listening to music were significantly higher compared to all other recovery activities (*p’s* < 0.001), except for watching a TV series, sleeping, and talking to a partner/significant other. This suggests that participants strongly value music listening as an activity to unwind after a stressful situation, to a similar extent as watching TV, sleeping, and talking to significant others. Post-hoc comparisons between other recovery activities are available at the study’s OSF page (https://osf.io/9pxhj).

**Table 2:** Means and standard deviations of recovery activity choices

Recovery activity	*m*	*SD*	Post-hoc comparison with “Listening to self-selected music”	
			*t (df)*	*p*
Listening to self-selected music	78.46	23.28	–	–
Watching a TV series	74.06	24.21	−2.92 (468)	.488
Sleeping	72.78	27.86	−3.56 (469)	.054
Talking to a partner/significant other	72.38	29.69	−3.47 (467)	.076
Watching a movie	72.00	24.49	−4.67 (469)	< .001*
Talking to friends	70.42	26.93	−5.24 (467)	< .001*
Eating	68.84	27.25	−6.18 (469)	< .001*
Light physical exercise	58.89	29.35	−11.67 (469)	< .001*
Talking to family	56.34	31.28	−12.68 (469)	< .001*
Reading a book	53.47	31.62	−15.11 (468)	< .001*
Mindfulness	48.83	32.36	−18.30 (469)	< .001*
Sports/heavy physical exercise	46.18	33.05	−17.10 (468)	< .001*
Praying	38.65	39.40	−20.27 (469)	< .001*
Listening to the radio	36.67	31.22	−25.26 (469)	< .001*
Meditating	36.14	33.55	−24.49 (269)	< .001*
Drinking alcoholic beverages	31.76	33.33	−25.30 (468)	< .001*
Playing a musical instrument	30.66	32.93	−28.62 (467)	< .001*

*Statistically significant

#### Reasons for listening to self-selected music after stress

**Fig. 1:**
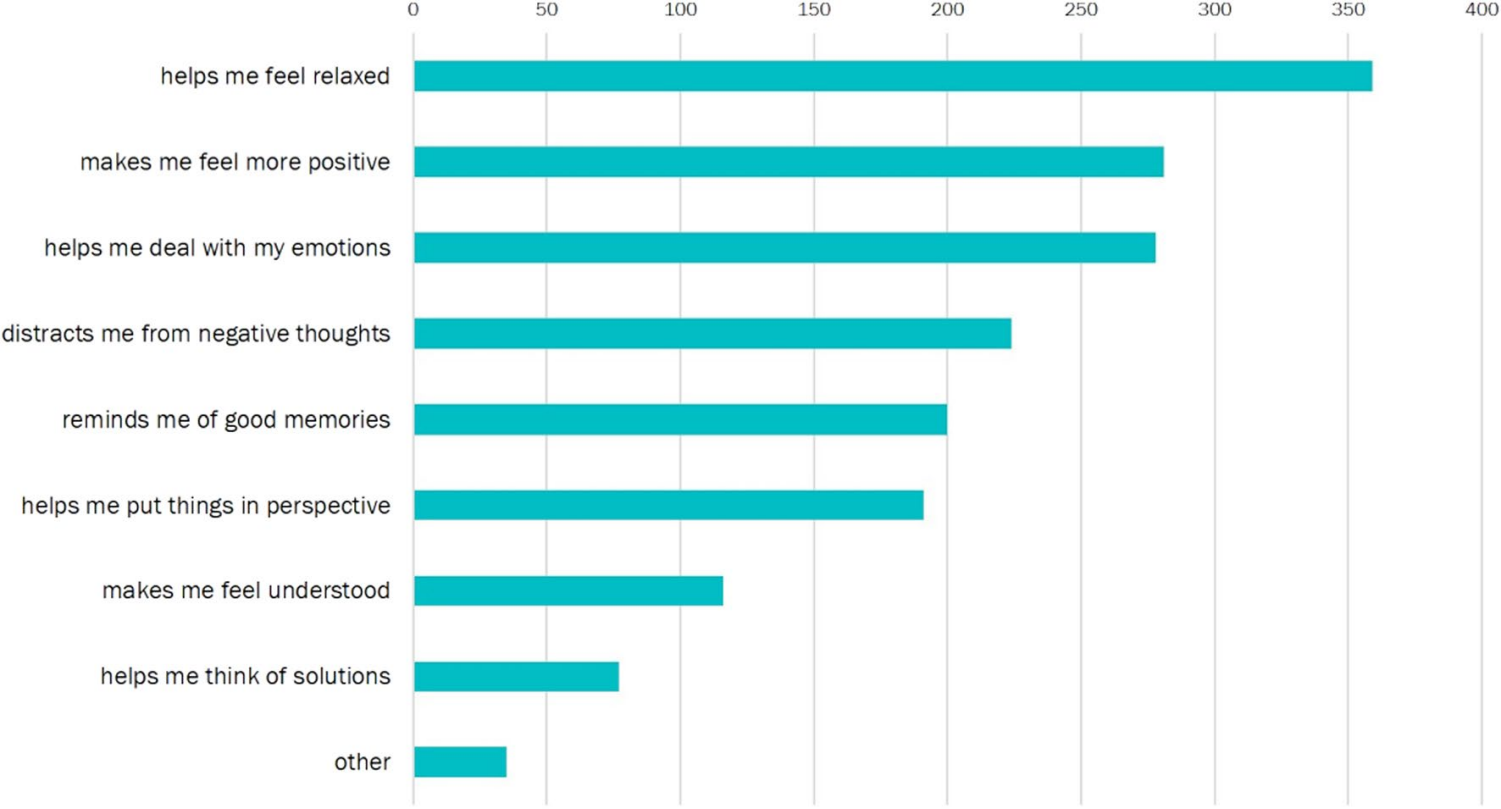
Cumulative frequencies of reasons for listening to self-selected music after stress. As participants could provide multiple responses, the number of responses in this measure is larger than the number of participants in our sample

Figure 1 summarizes response frequencies for each music listening reason (*N*_*responses*_ = 1761). The most selected reason for listening to music after a stressful situation was that music helped participants feel relaxed (*n*_*relaxation*_ = 359 [20.4%]). This suggests participants predominantly sought to use music listening to self-regulate their emotions through relaxation.

#### Characteristics of self-selected stress recovery music

**Fig. 2:**
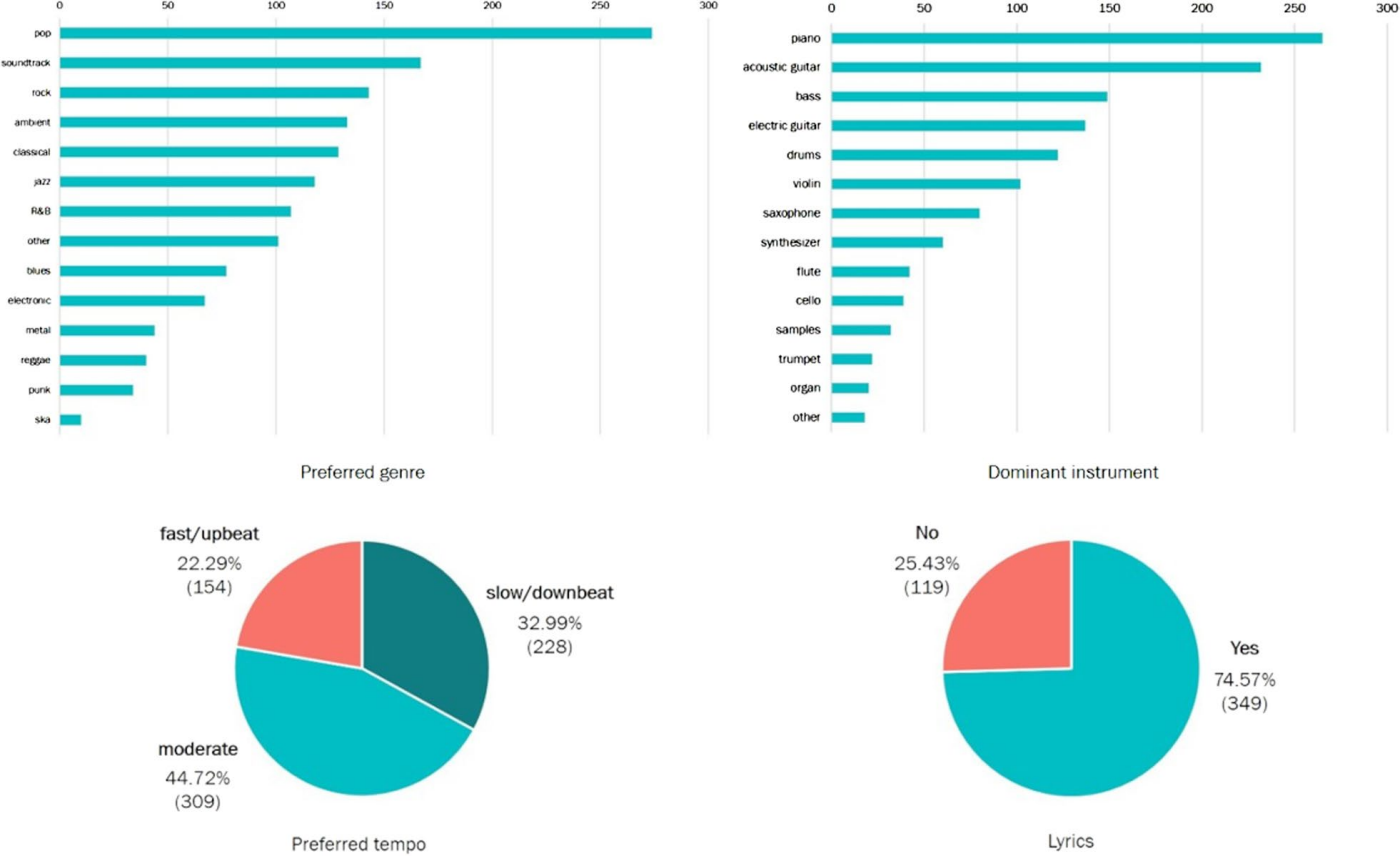
Characteristics of participants’ self-selected music for stress recovery. (top left) Cumulative frequencies for choice of music genre. (top right) Cumulative frequencies for dominant instrument. (bottom left) Cumulative frequencies for perceived tempo. (bottom right) Cumulative frequencies for whether the music should contain lyrics. Given that participants could provide multiple responses, the number of responses in all measures is larger than the number of participants in our sample

Figure 2 summarizes participants’ description of the music they would listen to after stress. Overall, participants described stress recovery music to be ‘pop’ music with moderate tempo, which had piano as the dominant instrument. Participants (74.57%) preferred the music to be lyrical, and reported that the lyrics should convey a story, be about love or positivity, or anything so long as they were able to sing along with the music.

#### Emotional responses to self-selected stress recovery music

**Fig. 3:**
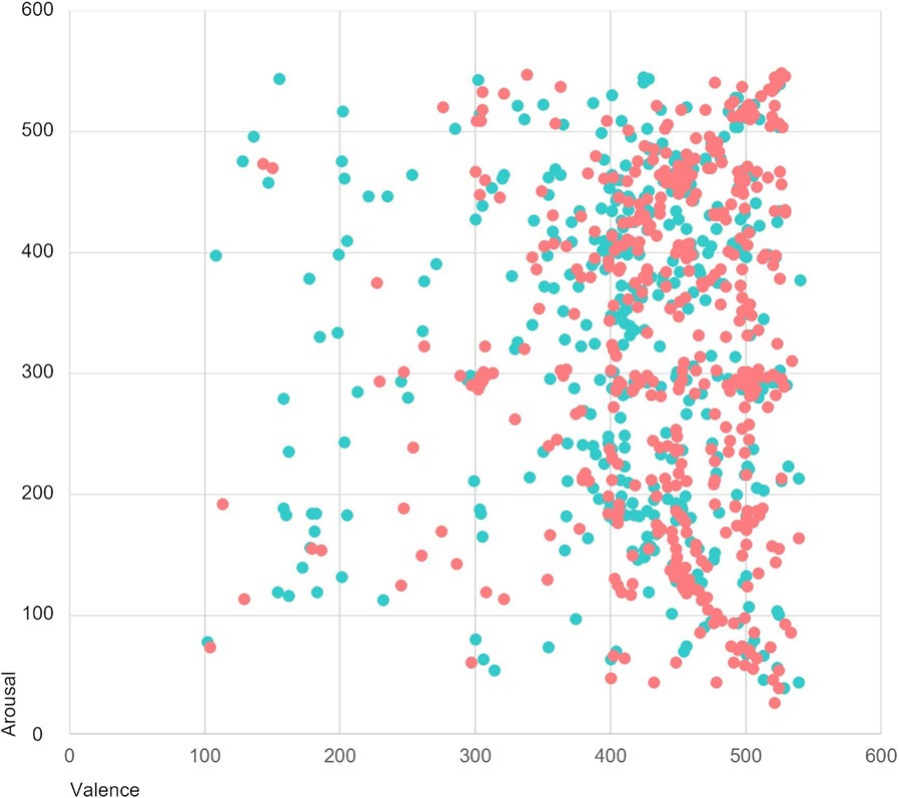
Desired perceived (cyan) and felt (red) emotions to self-selected music for stress recovery

Table 3 provides a summary of means and standard deviations of participants’ desired perceived emotional valence (PEV), perceived emotional arousal (PEA), felt emotional valence (FEV), and felt emotional arousal (FEA) to their self-selected music. Figure 3 provides a visual representation of these variables in two-dimensional space. On average, desired perceived emotions to self-selected music for stress recovery were characterized by positive valence and moderate arousal (Table 3). We obtained 389 self-reported descriptions of desired perceived emotions, which included, for example “calm”, “happiness”, “comfort”, and “relaxation.” Desired felt emotions were similarly characterized by positive valence and moderate arousal (Table 3). We obtained 338 self-reported descriptions of desired felt emotions, which included, for example, “calm”, “happiness”, “relaxation”, and “relief.”

**Table 3:** Means and standard deviations of desired emotions to self-selected music

	*m*	*SD*
Valence		
Perceived valence (PEV)	414.55	87.37
Felt valence (FEV)	439.97	73.63
Arousal		
Perceived arousal (PEA)	294.79	117.55
Felt arousal (FEA)	286.30	124.95

To further examine differences between perceived and felt emotions to self-selected music, within-subjects *t*-tests were conducted between PEV and FEV, and between PEA and FEA. The tests reveal a statistically significant difference between PEV and FEV mean scores (*t*[465] = 49.01, *p* < 0.001), but not between PEA and FEA. Overall, these results suggest that following a stressful situation, participants desired to listen to music which conveyed emotions with positive valence and moderate arousal, with the desire to experience emotions with more positive valence and similar arousal afterwards.

### Audio feature commonalities of self-selected stress recovery songs

**Fig. 4:**
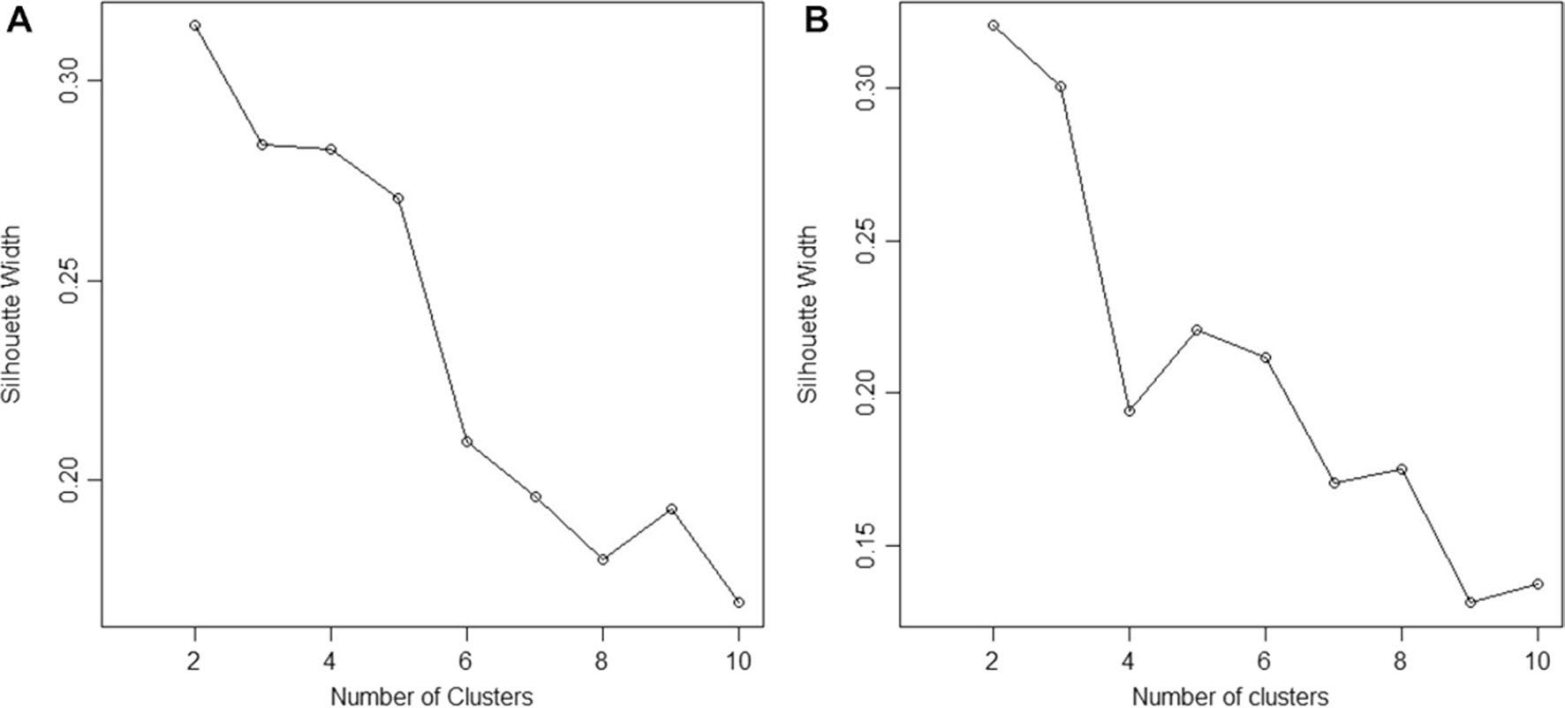
Plot of silhouette widths for *k* = 2–10 in the exploratory (**A**) and confirmatory (**B**) subsets

A cluster analysis was conducted to determine audio feature commonalities between self-selected stress recovery songs. In line with our split-sample approach [61, 62], the cluster analysis was first conducted in an exploratory data subset, before being reproduced in the confirmatory data subset. First, based on an examination of silhouette widths for *k* = 2–10 in the exploratory subset, *k* = 2 was selected as the optimal number of clusters as it resulted in the highest silhouette width value. Similarly, an examination of silhouette widths also supported *k* = 2 in the confirmatory subset (Fig. 4).

**Fig. 5:**
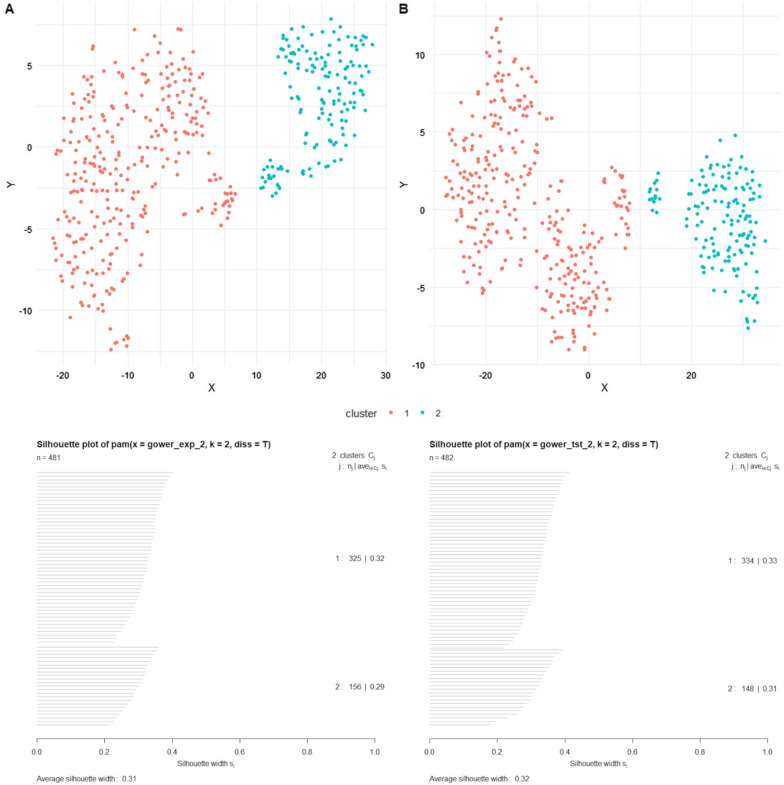
Cluster analysis results in the exploratory (column** A**) and confirmatory (column** B**) subsets. The figure shows *k* = 2 clusters plotted in two-dimensional space (top) and silhouette plots for both clusters (bottom)

Next, a *k*-medoid cluster analysis was conducted in the exploratory and confirmatory subsets with Spotify audio features key, mode, time signature, acousticness, danceability, energy, instrumentalness, liveness, loudness, speechiness, musical valence, and tempo as clustering variables (Fig. 5). Table 4 provides a summary of clustering variable means in both exploratory and confirmatory subsets.

**Table 4:** Clustering variable means in exploratory and confirmatory subsets

Audio feature	Exploratory subset			Confirmatory subset		
	Cluster 1	Cluster 2		Cluster 1	Cluster 2	
	*m (SD)*	*m (SD)*	Sig. test	*m (SD)*	*m (SD)*	Sig. test
Danceability	.52 (.17)	.56 (.18)	*t*(288) = −2.50, *p* = .012^†^	.53 (.17)	.56 (.16)	*t*(287) = −1.93, *p* = .054^††^
Energy	.54 (.26)	.59 (.23)	*t*(338) = −1.97, *p* = .050^††^	.53 (.26)	.59 (.25)	*t*(294) = −2.77, *p* = .005^†^
Key	5.15 (3.43)	4.98 (3.64)	*t*(290) = .49, *p* = .620	4.66 (3.64)	5.59 (3.46)	*t*(295) = −2.70, *p* = .007^†^
Loudness	−8.75 (5.22)	−8.44 (4.63)	*t*(340) = −.66, *p* = .512	−8.96 (5.04)	−8.55 (5.67)	*t*(255) = −0.76, *p* = .451
Mode	1 (−)	0 (−)	*χ*^*2*^(1) = 476.45, *p* < .001^†^	1 (−)	0 (−)	*χ*^*2*^(1) = 477.31, *p* < .001^†^
Speechiness	.06 (.05)	.07 (.07)	*t*(240) = −1.95, *p* = .052^††^	.06 (.06)	.08 (.08)	*t*(216) = −2.52, *p* = .012^†^
Acousticness	.39 (.36)	.35 (.33)	*t*(325) = 1.19, *p* = .235	.41 (.35)	.33 (.33)	*t*(295) = 2.34, *p* = .020^†^
Instrumentalness	.12 (.28)	.18 (.33)	*t*(265) = −1.96, *p* = .051^††^	.11 (.26)	.16 (.32)	*t*(241) = −1.58, *p* = .115
Liveness	.18 (.14)	.19 (.16)	*t*(274) = −.74, *p* = .461	.18 (.14)	.19 (.15)	*t*(270) = −.62, *p* = .536
Musical valence	.38 (.23)	.43 (.25)	*t*(286) = −1.93, *p* = .054^††^	.38 (.23)	.39 (.22)	*t*(297) = −.48, *p* = .634
Tempo	120.61 (31.72)	119.11 (28.56)	*t*(336) = .52, *p* = .603	119.94 (29.35)	117.70 (28.93)	*t*(285) = .78, *p* = .434
Duration*	4.09 (1.14)	4.29 (1.76)	*t*(219) = −1.33, *p* = .183	4.09 (1.46)	4.05 (1.58)	*t*(263) = .26, *p* = .794
Time signature	3.89 (.40)	3.93 (.40)	*t*(309) = −1.04, *p* = .298	3.89 (.37)	3.97 (.27)	*t*(373) = −2.56, *p* = .011^†^

Sig. test = significance test; * = in minutes; ^†^ = statistically significant at *p* = .05; ^††^ = statistically significant at *p* = .10

In the exploratory subset, mean audio feature differences suggest that self-selected songs in cluster 1 and cluster 2 were distinguished by danceability and mode. Overall, songs in cluster 1 were less danceable compared to songs in cluster 2. Further, songs in cluster 1 were predominantly in major mode while songs in cluster 2 were in minor mode. Mean audio feature differences also suggest that songs in cluster 1 may potentially be distinguished from those in cluster 2 by energy, speechiness, instrumentalness, and musical valence. However, cutoff scores for speechiness (< 0.33 = music only) and instrumentalness (> 0.5 = instrumental) in the Spotify Data Catalogue (SDC; Table 1) imply that differences in these features may not necessarily be meaningful. Thus, based on these differences, there may be a tendency for songs in cluster 1 to also be calmer and sadder compared to songs in cluster 2.

In the confirmatory subset, mean audio feature differences between clusters partially reproduced the pattern identified in the exploratory subset. Self-selected songs in cluster 1 and cluster 2 of the confirmatory subset were similarly distinguished by danceability, mode, energy, and speechiness, but not by instrumentalness and musical valence. Furthermore, songs in cluster 1 and cluster 2 in the confirmatory subset could be distinguished by key, acousticness, and time signature. However, as the SDC interprets time signature values of 3.89 and 3.97 as ‘4/4’, the between-cluster difference in time signature may be negligible. Thus, based on these differences, self-selected songs in cluster 1 of the confirmatory subset tended to be less danceable, calmer, and contained more acoustic instruments compared to songs in cluster 2. Further, songs in cluster 1 of the confirmatory subset were predominantly in the key of E and major mode compared to songs in cluster 2, which were mostly in F and minor mode. As in the exploratory subset, between-cluster differences in speechiness were not considered meaningful.

In summary, based on consistent mean audio feature differences in the exploratory and confirmatory subsets, self-selected songs for the purpose of stress recovery can be categorized into two clusters, which are distinguished by danceability, mode, and energy. The first cluster (i.e., the ‘calm major mode’ cluster) included songs in major mode that were less danceable and calmer compared to songs in the second cluster (i.e., the ‘moderate minor mode’ cluster). Songs in the 'calm major mode’ cluster include, for example, “Memories” by Maroon 5, “Good Times, Bad Times” by Led Zeppelin, and “The Long and Winding Road” by The Beatles. Meanwhile, songs in the ‘moderate minor mode’ cluster include, for example, “Shape of You” by Ed Sheeran, “Northern Lights” by Ola Gjeilo, and “Keep Your Head Up” by Ben Howards. Despite differences in danceability, mode, and energy, songs in the’calm major mode’ and ‘moderate minor mode’ clusters shared commonalities in key, loudness, speechiness, acousticness, instrumentalness, liveness, musical valence, tempo, duration, and time signature. Specifically, songs in both clusters were perceptually quiet, contained no spoken words, were not acoustic, not instrumental, nor performed live. Songs in both clusters were also mostly in the key of E, were performed with moderate tempo in 4/4, and conveyed low-to-moderate valence.

### Song audio features and emotional states

Multiple regression analyses in the exploratory and confirmatory data subsets were conducted to investigate the relation between Spotify audio features and participants’ desired emotional responses to self-selected music listening. In the exploratory subset, the overall regression model for PEA was statistically significant (*F*[13, 464] = 4.59, *p* < 0.001; *adj. R*^*2*^ = 8.91%), suggesting that higher instrumentalness was associated with lower PEA (*β* = -0.21, *p* < 0.001). Next, the overall regression model for FEA was statistically significant (*F*[13, 463] = 8.56, *p* < 0.001; *adj. R*^2^ = 18.16%), indicating that higher PEA scores (*β* = 0.41, *p* < 0.001), higher loudness (*β* = 0.22, *p* = 0.005), and higher speechiness (*β* = 0.09, *p* < 0.044) were associated with higher scores for FEA. With regards to emotional valence, the overall regression model for perceived emotional valence (PEV) did not reach statistical significance (*F*[13, 464] = 0.87, *p* = 0.588). Meanwhile, the overall model for FEV was statistically significant (*F*[13, 463] = 18.54, *p* < 0.001; *adj. R*^*2*^ = 33.98%), suggesting that higher PEV scores (*β* = 0.59, *p* < 0.001) and lower acousticness (*β* = −0.14, *p* = 0.027) were associated with higher FEV scores.

In the confirmatory subset, the overall regression model for PEA was statistically significant (*F*[13, 467] = 4.41, *p* < 0.001; *adj. R*^*2*^ = 8.46%), suggesting that higher energy (*β* = 0.25, *p* = 0.018) and minor mode (*β* = -0.11, *p* = 0.021) were associated with higher scores for PEA. Next, the overall model for FEA was statistically significant (*F*[13, 466] = 13.64, *p* < 0.001; *adj. R*^*2*^ = 26.93%), indicating that higher PEA scores (*β* = 0.52, *p* < 0.001) and lower tempo (*β* = −0.09, *p* = 0.027) were associated with higher scores for FEA. With regards to emotional valence, the overall model for PEV did not reach statistical significance (*F*[13, 467] = 0.74, *p* = 0.728). On the other hand, the overall model for FEV was statistically significant (*F*[13, 466] = 14.97, *p* < 0.001; *adj. R*^2^ = 28.95%), with only higher PEV scores being associated to higher FEV scores (*β* = 0.55, *p* < 0.001).

The results of the regression analyses in the confirmatory subset do not fully reproduce those in the exploratory subset. Taken together, the pattern of results in the exploratory and confirmatory data subsets suggests that no individual audio features were associated with participants’ desired emotions both during and after listening to music. Instead, participants’ desired emotional valence (i.e., felt emotional valence) and arousal (i.e., felt emotional arousal) after listening to music was associated with their desired emotional valence (i.e., perceived emotional valence) and arousal (i.e., perceived emotional arousal) while listening to music.

## Discussion

The present study attempted to: (a) investigate which recovery activities are chosen by individuals for the purpose of stress recovery; (b) identify musical audio feature commonalities between songs self-selected by participants for the purpose of stress recovery; and (c) investigate the relationship between musical audio features and desired emotional responses following stress. An exploratory cross-sectional survey revealed that, among a selection of activities, participants rated music listening, watching TV, sleeping, and talking to significant others as activities they are most likely to engage in for the purpose of stress recovery. Cluster analyses of participants’ self-selected stress recovery songs revealed two clusters of music that shared similarities in key, loudness, speechiness, acousticness, instrumentalness, liveness, musical valence, tempo, duration, and time signature. The two clusters were distinguished by danceability, energy, and mode, with a ‘calm major mode’ cluster comprising of major mode songs that were less danceable and less energetic compared to the minor mode songs that made up the ‘moderate minor mode’ cluster. Finally, multiple regression analyses revealed no significant associations between musical audio features and participants’ desired emotional responses to self-selected music listening in the context of stress recovery.

### Music listening as a stress recovery activity

The present study is among the first to demonstrate that individuals consider music listening a beneficial activity for stress recovery. Among several stress recovery activities [2, 10], our study suggests that participants valued music listening to a similar extent as watching TV, sleeping, and talking to significant others for the purpose of stress recovery. Should participants choose to listen to music, our study further showcases that the principal goal underlying this choice was to pursue relaxation. This finding is in line with previous research by Linnemann and colleagues [44], where participants reported lower perceived stress when listening to music for the purpose of ‘relaxation’ compared to when they listened to music for other purposes, such as ‘activation’ or ‘distraction. Though we were unable to directly relate participants’ self-selected music to stress recovery in our current study, our findings provide circumstantial support to the popular notion that music listening may be a valuable and accessible means to facilitate stress recovery [18, 21, 68].

Besides relaxation, participants also reported listening to music to experience positive emotions, cope with negative emotions, and distract themselves from negative thoughts after a (hypothetical) stressful situation. This finding lends support to notion that individuals use music listening as a strategy to self-regulate their emotions [43, 57, 69]. Interestingly, our findings show that despite differences in emotion regulation goals, participants selected songs with the desire to perceive emotions with positive valence and moderate arousal. Further, participants did so with the desire to feel emotions with higher positive valence, relative to the perceived emotion, with no change in arousal. This pattern is similar to that of previous studies, which demonstrated that individuals who listened to music that was congruent with their emotional self-regulation goals experienced (i.e., felt) emotions with higher emotional valence and lower emotional arousal, compared to individuals who listened to music that was incongruent with their goals [39–44]. It is thus plausible to assume that changes in emotional valence may play an important role in emotional self-regulation, with perceived emotions in self-selected music acting as a steppingstone to improve an individual’s felt emotions. This process may in turn be beneficial to stress recovery. In line with the broaden-and-build theory of positive emotions [70], positive emotional experiences (through successful emotional self-regulation) may help build resilience over time, which may facilitate recovery from future or upcoming stressors [9].

### Audio feature commonalities between self-selected stress recovery music

Classical music is often considered the standard with which studies on music listening and stress are conducted [71]. Indeed, classical music is heavily featured in various ‘relaxation’ tapes and online playlists, and excerpts from such compilations have been previously used in research on music listening and stress recovery [18, 68, 72]. Against this practice, the present study shows that participants from diverse nationalities (see Additional file 1: Appendix A) selected “pop” as the musical genre they would prefer listening to following stress. More importantly, the present study showcases that focusing on musical audio features, as opposed to conventional musical genres, may yield a more nuanced comparison of songs for the purpose of stress recovery:

First, the results of our cluster analyses suggest that participants’ self-selected stress recovery songs could be categorized into a ‘calm major mode’ or a ‘moderate minor mode’ cluster, based on their danceability, energy, and musical mode. Regardless of cluster membership, self-selected stress recovery songs shared commonalities in key, loudness, speechiness, acousticness, instrumentalness, liveness, musical valence, tempo, duration, and time signature. This suggests that the average self-selected stress recovery song is likely composed in the key of E, perceptually quiet, contains no spoken words (i.e., unlike a podcast or news segment), contains few acoustic instruments, contains lyrics, is not a live performance, conveys emotions with low-to-moderate valence, is neither fast nor slow, lasts approximately four minutes, and has a regular 4/4 structure. Interestingly, this description encompasses songs that would typically be categorized into different conventional genres, such as “Good Times, Bad Times” by Led Zeppelin (hard rock) and “Wake Me Up” by Avicii (electronic dance/electronic folk), “24–25” by Kings of Convenience (indie folk-pop), and “Don’t Look Back in Anger” by Oasis (British pop). Thus, we believe that describing stress recovery songs in terms of audio features may provide a more useful start to subsequent investigations into the effects of music listening on stress recovery, in contrast to comparing conventional musical genres or arbitrarily selecting ‘relaxing’ music.

Next, multiple studies on music listening and stress recovery have claimed that music with slow tempo, which commonly encompasses songs set to 60–80 bpm, would be most beneficial for stress recovery due to its similarity to the human resting heart rate [73–77]. By examining audio features, it is thus interesting to note that across both of our identified clusters, the average tempo of self-selected songs ranged between 119–122 bpm. This discrepancy may be partially explained by differences in human beat perception, as individuals are not always accurate at identifying beat, particularly in complex, syncopated rhythms [78–80]. Other studies claim that individuals simply prefer tempi that are slightly above their resting heart rate [81]. Presently, systematic investigations into the effect of musical tempo are still in their infancy [74, 76, 77], particularly those making use of self-selected music. Thus, the interplay between the various processes that are part of beat perception, along with their relation to the physiological stress response may be an interesting avenue for further research.

### Desired emotional responses to self-selected stress recovery music

The present study found no evidence of an association between Spotify audio features and participants’ desired emotional responses to self-selected music listening. Rather, perceived emotional valence was associated with an increase in felt emotional valence, while perceived emotional arousal was associated with an increase in felt emotional arousal. Our findings contrast those forwarded by studies in music emotion recognition (MER), which suggest that musical audio features, such as tempo, pitch, and timbre, may predict both perceived emotional valence and perceived emotional arousal [7, 29, 30]. A potential explanation for this difference is that the present study measured participants’ *desired* emotional responses to self-selected music, that is, participants’ expectations about the emotions they would perceive from the music, and the emotions they would feel afterwards. Perhaps, participants’ desired perceived and felt emotions may not accurately represent their actual perceived and felt emotions to music listening. Alternatively, it is possible that Spotify audio features may not have the same predictive power as the conventional audio features they are derived from, specifically with regards to (desired) emotional responses to music listening [46]. Though studies have previously leveraged Spotify audio features to distinguish “relaxing” and “non-relaxing” songs [59], identify audio feature commonalities in national top fifty songs [58], and predict global music listening behaviour [82], it remains unclear whether associations between Spotify audio features and emotional responses to music do exist.

### Limitations

First, to obtain a more comprehensive portrait of participants’ music listening behaviour following stress, several questionnaire items, such as choice of recovery activity and reasons for listening to music, allowed participants to select multiple responses. Our study has shown it to be realistic to provide participants to select multiple answer categories, as individuals simply have different recovery activity preferences and multiple reasons to listen to music. However, this decision has prevented us from exploring other potentially relevant relationships between our measured variables. For example, it was difficult to verify the supposed association between different reasons for listening to music and desired emotional responses to music, as it would create multiple within-subject dependencies that were difficult to statistically account for. To circumvent this limitation, each of these relationships could be investigated individually in future studies. For example, participants could be asked to listen to different songs selected for different purposes, following which their emotional reactions to these specific songs could be measured.

Second, the present study included no stress induction procedure, and no measure of actual emotional states while listening to music: participants were not instructed to listen to their self-selected music while being stressed, but instead had to report their expected (i.e., desired) emotional states to their self-selected songs after hypothetical stress. Given this limitation, we were not able to conclusively determine whether participants’ self-selected songs do, indeed, induce their self-reported perceived and felt emotions. Further, we were not able to conclusively determine whether our findings are generalizable to situations in which an individual is verily stressed and listens to self-selected songs. Future studies could circumvent this limitation by adopting an experimental design and having participants listen to the songs they have selected after a stressful experience, measuring participants’ emotional responses at different moments of interest. For example, following a stress induction procedure, participants’ emotional responses to self-selected music listening could be continuously measured over time (e.g., see Nagel et al. [56]), to better map how participants’ emotional responses to music unfold throughout a recovery period. Alternatively, to more reliably assess the relationship between music listening and stress recovery in ecologically valid settings, a study design similar to Linnemann and colleagues [44, 83] may be implemented, in which participants are asked to describe or report contextual factors related to their stressor and music listening behaviour.

### Directions for future research

Based on our findings, we propose two research lines that may ultimately allow us to further understand the recovery potential of music listening:

First, we believe future studies should further investigate the association between a song’s audio features with both psychological and physiological stress recovery outcomes. For this purpose, the audio feature clusters identified in the present study may be used as reference in selecting appropriate musical stimuli. The eventual goal of these studies would be to identify whether the effect of music listening on stress recovery can occur due to the music itself, while minimizing the influence of other variables such as preference, familiarity, or self- vs. experimenter-selection [8, 84].

Second, future studies should identify the various boundary, stimulating, and working mechanisms of the relationship between music listening and both psychological and physiological stress recovery. For example, prior studies have identified the potential role of musical preference [84] and musical familiarity [85]. However, future studies could further examine the role of emotion self-regulation strategies [69] and self-selection [8], as objectively ‘relaxing’ music may perhaps only be perceived as ‘relaxing’ when self-selected by individuals.

## Conclusion

Music listening is often portrayed as a promising method to promote stress recovery. However, since music listening is a highly personal activity, new approaches are needed to better discern the effects of music listening on the recovery process. The present study provides initial evidence that individuals do value music listening as an activity that helps them unwind after a stressful situation, with relaxation being the principal reason underlying the choices of self-selected music for stress recovery. Through an audio feature approach, the present study identifies two clusters of self-selected stress recovery music, that are distinguished by danceability, energy, and mode, yet share commonalities in key, loudness, speechiness, acousticness, instrumentalness, liveness, musical valence, tempo, duration, and time signature. Although the relationship between these audio features, emotional experiences, and stress recovery still warrants further research, the from the present study may serve as a catalyst for future research into the nuanced effects of music listening on stress recovery.

## Supplementary Information

**Additional file 1**. Complete list of participant nationalities.

## Data Availability

All data and materials relevant to this study are available on the study’s OSF page (https://osf.io/9pxhj/). These include the pre-registration document, the online questionnaire, datasets, and R code to replicate the study’ analyses. Datasets generated and analysed in the current study are also available from the corresponding author on request.

## Abbreviations

PEV: Perceived emotional valence
PEA: Perceived emotional arousal
FEV: Felt emotional valence
FEA: Felt emotional arousal
