# Supplementary material for: The sound of stress recovery: an exploratory study of self-selected music listening after stress
Source: BMC Psychol. 2023 Feb 10;11:40. doi: 10.1186/s40359-023-01066-w (PMC9912599; doi:10.1186/s40359-023-01066-w)
Supplement: Supplementary file 1 — Additional file 1. Complete list of participant nationalities. [file 40359_2023_1066_MOESM1_ESM.docx]

**Appendix A**

**Table 1**

*List of Participant Nationalities*

| Continent | Nationality | *n* (participants) | *N* (continent) |
| --- | --- | --- | --- |
| Asia | Indonesian | 202 | 211 |
|  | Chinese | 3 |  |
|  | Filipino | 2 |  |
|  | Israeli | 1 |  |
|  | Japanese | 1 |  |
|  | Malaysian | 1 |  |
|  | Russian | 1 |  |
| Africa | South African | 3 | 4 |
|  | Nigerian | 1 |  |
| Australia | Australian | 1 | 2 |
|  | New Zealander | 1 |  |
| Europe | Polish | 49 | 226 |
|  | Dutch | 37 |  |
|  | British | 35 |  |
|  | Portuguese | 30 |  |
|  | Italian | 25 |  |
|  | Greek | 10 |  |
|  | German | 9 |  |
|  | Finnish | 6 |  |
|  | Irish | 6 |  |
|  | Belgian | 3 |  |
|  | Slovenian | 3 |  |
|  | Spanish | 3 |  |
|  | French | 2 |  |
|  | Austrian | 1 |  |
|  | Croatian | 1 |  |
|  | Czech | 1 |  |
|  | Danish | 1 |  |
|  | Estonian | 1 |  |
|  | Latvian | 1 |  |
|  | Swedish | 1 |  |
|  | Welsh | 1 |  |
| North & South America | Mexican | 11 | 23 |
|  | American | 6 |  |
|  | Canadian | 2 |  |
|  | Chilean | 2 |  |
|  | Argentinian | 1 |  |
|  | Venezuelan | 1 |  |
| Multiple | Multiple | 4 | 4 |
